# The Antimicrobial Susceptibility of *Klebsiella pneumoniae* from Community Settings in Taiwan, a Trend Analysis

**DOI:** 10.1038/srep36280

**Published:** 2016-11-08

**Authors:** Wu-Pu Lin, Jann-Tay Wang, Shan-Chwen Chang, Feng-Yee Chang, Chang-Phone Fung, Yin-Ching Chuang, Yao-Shen Chen, Yih-Ru Shiau, Mei-Chen Tan, Hui-Ying Wang, Jui-Fen Lai, I-Wen Huang, Tsai-Ling Lauderdale

**Affiliations:** 1 Department of Internal Medicine, National Taiwan University Hospital, Taipei, Taiwan; 2 National Institute of Infectious Diseases and Vaccinology, National Health Research Institutes, Zhunan, Taiwan; 3 Graduate Institute of Clinical Pharmacy, College of Medicine, National Taiwan University, Taipei, Taiwan; 4 Division of Infectious Diseases and Tropical Medicine, Tri-Service General Hospital, Taipei, Taiwan; 5 Department of Internal Medicine, National Defense Medical Center, Taipei, Taiwan; 6 Division of Infectious Diseases, Department of Medicine, Taipei Veterans General Hospital, Taipei, Taiwan; 7 Department of Internal Medicine, Chi-Mei Medical Center, Tainan, Taiwan; 8 Department of Medicine, Kaohsiung Veterans General Hospital, Kaohsiung, Taiwan; 9 School of Medicine, National Yang Ming University, Taipei, Taiwan

## Abstract

Drug-resistant *Klebsiella pneumoniae*, especially extended-spectrum β-lactamase (ESBL)- and/or AmpC β-lactamase-producing strains, is an emerging problem worldwide. However, few data focusing on drug susceptibility of *K. pneumoniae* from community is available. In this study, we analyzed 1016 *K. pneumoniae* isolates from outpatients or those visiting emergency rooms collected during 2002–2012 from Taiwan Surveillance of Antimicrobial Resistance program. Significantly decreased susceptibilities to 3^rd^ generation cephalosporins and ciprofloxacin were found during the study period. By 2012, susceptibility to cefotaxime and ciprofloxacin was 83.6% and 81.6%, respectively. The prevalence of ESBL-producers increased from 4.8% in 2002 to 11.9% in 2012 (P = 0.012), while that of AmpC β-lactamase-producers increased from 0% to 9.5% in the same period (P < 0.001). Phylogenic analysis of the ESBL and AmpC-β-lactamase-producers by pulsed-field gel electrophoresis and multi-locus sequence typing revealed wide genetic diversity even among the most common sequence type 11 isolates (33.0%). By multivariate analysis, later study year, elderly, and urine isolates were associated with carriage of ESBL genes, while only urine isolates were associated with carriage of AmpC β-lactamase genes. Further studies are needed to determine which antibiotics are reasonable empirical therapy options for patients presenting with severe sepsis that might be caused by *K. pneumoniae*.

*Klebsiella pneumoniae* belongs to the family of *Enterobacteriaceae*. In addition to the ability to colonize gastrointestinal tract, nasopharynx, and skin, *K. pneumoniae* could cause various infection syndromes, including urinary tract infection, intra-abdominal infection, skin and soft tissue infection, and pneumonia, in both community and healthcare-associated settings^1^,^2^,^3^.

Treatment of bacterial infections depends heavily on effective antimicrobial therapy. Delayed use of effective antibiotics has been associated with a higher mortality rate in patients with severe infections^4^. Therefore, the presence of drug resistance in the infecting pathogen would adversely affect the treatment outcome^5^. One major drug resistance mechanism of concern in *K. pneumoniae* is the production of β-lactamases, especially extended-spectrum β-lactamases (ESBLs) and/or AmpC β-lactamases because these isolates are resistant to broad-spectrum cephalosporins and/or β-lactam/β-lactamase inhibitors^6^,^7^. In addition, these isolates are often resistant to several classes of non-β-lactam antibiotics.

The overall prevalence of ESBL-producing *K. pneumoniae* isolates varies widely in different studies, from 3.6% in Canada, 16% in U.S.A, to 26.2% in Korea, and 39.3% in Eastern Europe^8^,^9^,^10^,^11^,^12^. The Study for Monitoring Antimicrobial Resistance Trends (SMART) has shown that the prevalence of ESBL-producing *K. pneumoniae* isolates from intra-abdominal infection (IAI) was also high^13^. Of special concern also is that a trend of increased prevalence of ESBLs among *K. pneumoniae* has been observed globally, even in low prevalence countries such as Canada^14^. A similar trend has been noted in pediatric patients^15^,^16^. Data on the prevalence of AmpC β-lactamases carriage are less available, but an increased trend has also been observed from different studies^17^,^18^. Community-acquired ESBL *K. pneumoniae* infection has also emerged. One study from France showed that ESBL-producing strains accounted for 6.6% of community-onset *K. pneumoniae* urinary tract infections^19^. It has been recognized that community-based patients can be reservoirs for ESBL- and AmpC β-lactamase-producing strains, especially when they are from nursing home or clinics^20^.

In Taiwan, nosocomial ESBL *K. pneumoniae* infection has been a recognized emerging threat^21^,^22^. However, updated epidemiological and microbiological data about *K. pneumoniae* from community settings in Taiwan are still limited. Such data could impact empirical therapy regimen. The present study analyzed data on *K. pneumoniae* from community settings collected by the Taiwan Surveillance of Antimicrobial Resistance (TSAR) program from 2002 to 2012 with the goals of providing the aforementioned valuable information to update the suggestion of empirical antibiotics regimen.

## Result

Between 2002 and 2012, 1016 non-duplicated *K. pneumoniae* isolates from TSAR III to VIII were included. The number of isolates from each study period was as follows: TSAR III (2002): 124, IV (2004): 149, V (2006): 152, VI (2008): 186, VII (2010): 195, VIII (2012): 210. A total of 37.2% (378) of the isolates were from blood samples, and 30.4% (309) were from urine. The remaining isolates were grouped as others (32.4%, 329). The mean age of the source patients was 60.6 ± 21.2 years, with age data missing in 20 people. The percentage of adults (19–64 y) and elderly (≥65 y) was 45.2% (450) and 49.2% (490), respectively. The proportion of pediatric patients (≤18y) was only 5.6% (56).

### Antimicrobial susceptibilities of *K. pneumoniae* over study periods

For ease of comparison, we grouped TSAR III (2002) ~V (2006) as Period I (total isolates number = 425) and TSAR VI (2008) ~ VIII (2012) as Period II (total isolates number = 591). The susceptibilities of *K. pneumoniae* to different antimicrobial agents, including β-lactams and non-β-lactams, are listed in Table 1. Decreased susceptibilities from Period I to Period II were noted in most of the antimicrobial agents tested. The most significant decrease was observed in all 1^st^, 2^nd^, and 3^rd^ -generation cephalosporins. However, 90.6% of isolates in Period I and 87.1% of isolates in Period II remained susceptible to amoxicillin/clavulanate. The susceptibilities to four different carbapenems were all well above 95%. Among non-β-lactams, significant decrease in susceptibility was observed in ciprofloxacin, and the susceptibilities to ciprofloxacin and levofloxacin in Period II were only 81.6% and 80.8%, respectively. Although the data was not available in period I, susceptibility to tigecycline was high (97.8%) in Period II.

**Figure 1:**
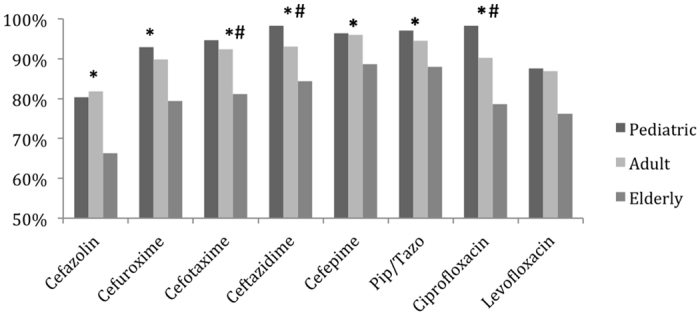
Susceptibilities of *K. pneumoniae* isolates to different antimicrobial agents among pediatric, adult, and elderly patients. ^*^The susceptibility is significantly lower among isolates from elderly compared with those from adult patients. (P < 0.05). ^#^The susceptibility is significantly lower among isolates from elderly compared with those from pediatric patients. (P < 0.05).

*K. pneumoniae* isolates from blood showed similar susceptibility pattern to those from specimens other than blood and urine (Table 2). However, isolates from urine were more resistant. For all cephalosporins and ciprofloxacin, the differences were significant. Among different age groups, isolates from elderly patients universally had higher rates of non-susceptibility compared to those from adult patients. For isolates from pediatric patients, rates of non-susceptibility were usually similar to or lower than those of adult patients, and all were lower than those of elderly patients (Fig. 1).

### Prevalence and susceptibility pattern of ESBL/AmpC β-lactamase-producers

A total of 138 isolates with aztreonam, ceftazidime, and/or cefotaxime MIC ≥ 2 mg/L were identified. All of them were subject to the CLSI ESBL phenotypic confirmatory test and detection of ESBL and AmpC β-lactamase genes by PCR. Among them, 27 isolates were positive for both ESBL and AmpC β-lactamase genes, 54 were positive for ESBL genes but negative for AmpC β-lactamase genes, 34 were positive for AmpC β-lactamase genes but negative for ESBL genes, and the remaining 23 isolates (901 isolates if those 878 with negative ESBL screening test are also pooled together) were negative for both ESBL and AmpC β-lactamase genes. No isolate with carbapenemase was detected. Rates of susceptibility were highest in isolates negative for ESBL and AmpC β-lactamase genes, followed by those with either ESBL or AmpC β-lactamase genes, and lowest in isolates carrying both ESBL and AmpC β-lactamase genes (Table 3). Of note, susceptibility to cefepime was significantly different between ESBL-positive group and ESBL-negative group, regardless of their AmpC β-lactamase gene status. Combining susceptible (S) and susceptible dose dependent (SDD) categories together, the susceptibility of cefepime was 99.9% in ESBL negative/AmpC β-lactamase negative group, but it was only 37% in ESBL-positive/AmpC β-lactamase-negative group. Co-resistance to aztreonam and ciprofloxacin could be observed among ESBL- and/or AmpC β-lactamase-producing strains. In addition, decreased susceptibility to carbapenems occurred among isolates with both ESBL and AmpC β-lactamase genes.

**Figure 2:**
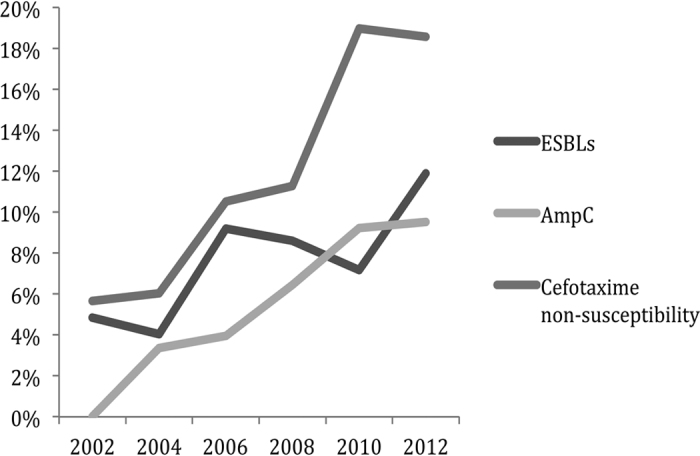
Increase of ESBL carriage, AmpC β-lactamase carriage, and cefotaxime non-susceptibility in *K. pneumoniae* from community settings, 2002–2012.

Between 2002 and 2012, ESBL-producers among our *K. pneumoniae* isolates increased from 4.8% to 11.9%, and AmpC β-lactamase-producers increased from 0% to 9.5%, both with p values of <0.05 in trend analysis (Table 4). The rate of cefotaxime non-susceptibility increased significantly from 5.7% to 18.7% during the study period (Fig. 2). Using cefotaxime non-susceptibility as predictors of ESBL- and/or AmpC β-lactamase-producers, the sensitivity, specificity, positive predictive value (PPV) and negative predictive value (NPV) was 98.3%, 98.2%, 87.6%, and 99.8%, respectively. If ceftazidime was used instead, then the sensitivity, specificity, PPV and NPV was 84.5%, 98.7%, 89.0%, and 98.5%, respectively.

Among 81 ESBL-producers identified, 52 (64%) carried CTX-M-type genes, 24 (30%) carried SHV-type genes, and 5 (6%) carried both CTX-M-type and SHV-type genes. For AmpC β-lactamase-producers, 90% (55 out of 61) carried DHA-type genes, and 10% (6 out of 61) carried CMY-type genes. CTX-M plus DHA genes were the most common combination for isolates with both ESBL and AmpC β-lactamase genes, followed by the combination of SHV and DHA genes, at 59% and 41%, respectively.

### Phylogenetic analysis of the ESBL and/or AmpC β-lactamase-producers

**Figure 3:**
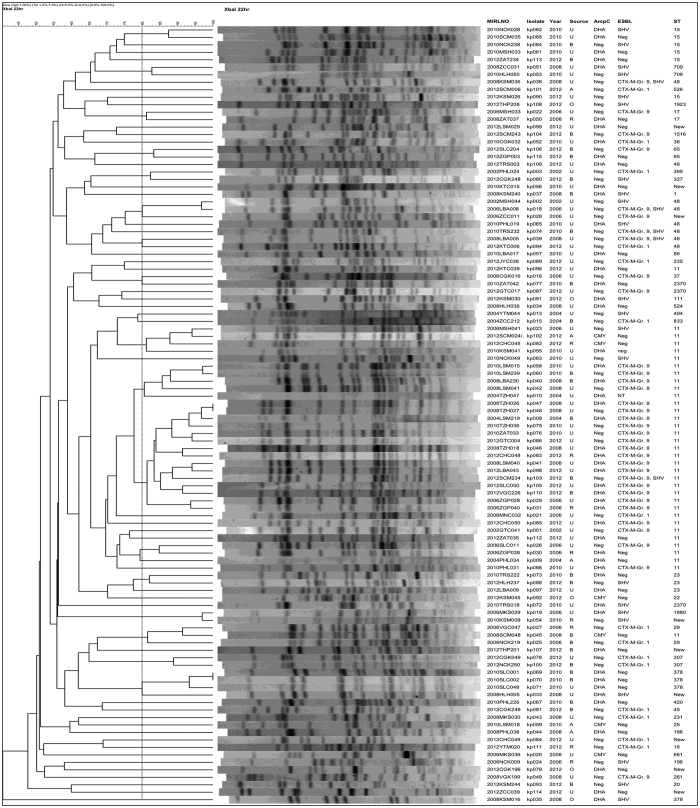
Dendrogram of XbaI-digested genomic DNA of *Klebsiella pneumoniae* isolates with ESBL and/or AmpC β-lactamase genes. *PFGE of 11 of the 115 isolates failed PFGE. The ST of these 11 isolates are ST11 (3), ST48 (2), and 1 each of ST23, ST48, ST65, ST378, ST750, ST897; Source: U, urine; B, blood; O, others. New ST, isolates with new allele profiles not in the MLST database.

The phylogenetic analysis of the ESBL and/or AmpC β-lactamase-producers was determined by multi-locus sequence typing (MLST) and pulsed-filed gel electrophoresis (PFGE). Among the 115 isolates, the most prevalent sequence types (STs) was ST11 (38, 33.0%), followed by ST48 (10, 8.7%), ST15 (6, 5.2%), and ST378 (6, 5.2%). Another 45 isolates belonged to 36 additional STs, and the remaining 10 isolates belonged to new STs. PFGE results demonstrated great genetic diversity with only one small cluster of 15 isolates sharing ≥ 80% similarity in PFGE pattern (Fig. 3). Although these 15 isolates all belonged to ST11, they were recovered from 11 hospitals (data not shown) in different study years and some carried different ESBL and/or AmpC β-lactamase genes combinations, and only 2 isolates from 2008 had indistinguishable PFGE patterns but one isolate carried CTX-M-type ESBL, while the other carried CTX-M-type ESBL and DHA-type AmpC-β-lactamase genes.

### Factors associated with carriage of ESBL and/or AmpC β-lactamase genes

In univariate analysis, study years, age groups, and specimen types were associated with carriage of ESBL and/or AmpC β-lactamase genes. By multivariate analysis, study year (2004 vs. 2012), age group (elderly vs. pediatric), and specimen types (urine vs. blood or others) remained factors associated with carriage of ESBL genes, while only specimen types (urine vs. blood or others) was associated with carriage of AmpC β-lactamase genes (Table 5).

## Discussion

The present study analyzed data on *K. pneumoniae* from a biennial nationwide surveillance program between 2002 and 2012 in Taiwan focusing on community-sourced isolates. Our data revealed decreased susceptibilities to most β-lactam antibiotics and fluoroquinolones during 2008–2012 (Period II) compared with those during 2002–2006 (Period I). For period II, susceptibilities to 1^st^, 2^nd^, and 3^rd^ generation cephalosporins were all lower than 90%, and the susceptibility to ciprofloxacin was 81.6%. Urine isolates in the present study tended to be less susceptible to most antibiotics than blood isolates. Furthermore, compared with studies focusing on community-acquired *K. pneumoniae* urinary tract infections from other countries, lower susceptibility of urine isolates was also observed in our study. For example, the susceptibility of our urine isolates was 79.3% to cefotaxime (92.8% in USA), 85.7% to cefepime (94% in U.S.A., and 97.7% in Japan), 84.4% for piperacillin-tazobactam (91% in U.S.A., and 97.7% in Japan), and 73.0% for ciprofloxacin (91.6% in U.S.A., 77.3% in Korea, and 91.7% in Japan.)^23^,^24^,^25^.

In our study, the overall percentage of ESBL-producing *K. pneumoniae* isolates was 7.6% but the prevalence of ESBL-producers increased significantly over the study years, from 4.8% in 2002 to 11.9% in 2012. In other countries, the prevalence of ESBL-producers was 7.2% among *K. pneumoniae* urine isolates causing community-acquired urinary tract infection in the United States^23^, 3.3% for community-onset *K. pneumoniae* bacteremia in Korea^26^, and only 3.6% in Canada even when both community-acquired and hospital-acquired infection isolates were included^8^. Compared with the studies aforementioned, our study revealed that the prevalence of ESBL-producers among community-acquired *K. pneumoniae* infections was higher in Taiwan. Furthermore, the present study also showed an increasing prevalence of AmpC β-lactamase-producers (from 0% in 2002 to 9.5% in 2012). *K. pneumoniae* carrying ESBL and/or AmpC β-lactamase genes usually also had higher rates of resistance to non-β-lactam antibiotics. For example, susceptibility to amikacin was 64.8% in isolates carrying ESBL genes and 67.7% in isolates carrying AmpC β-lactamase genes, compared to 99.6% in isolates with neither ESBL nor AmpC β-lactamase genes. Similarly, susceptibility to ciprofloxacin was 33.3% and 38.2% in isolates with ESBL and AmpC β-lactamase genes, respectively, while it was 92.5% in isolates with neither. For isolates with both ESBL and AmpC β-lactamase genes, susceptibility to amikacin and ciprofloxacin was only 25.9% and 0%, respectively, at rates similar to those previously reported^23^,^27^.

The high ciprofloxacin resistance rate among ESBL-positive or AmpC β-lactamase-positive isolates is alarming. Of special concern is that an earlier study conducted between 1998 and 2000 in Taiwan demonstrated that the ciprofloxacin resistance rate among ESBL-producing *K. pneumoniae* was 18.5%, which is much lower than that shown in our present study (77.8%)^28^. Because fluoroquinolones are important alternative antibiotics to carbapenems, and they are the antibiotics that have oral forms for treating ESBL-producers, the increased fluoroquinolone resistance raises great clinical concern. Kang and colleagues reported that prior use of fluoroquinolones could be an important independent risk factor for ciprofloxacin resistance in ESBL-producing *K. pneumoniae*^29^. Fluoroquinolone consumption in Taiwan has increased over the years^30^. From our study, overall susceptibility to ciprofloxacin was still high among pediatric patients (98.2%) but was only 78.6% among elderly patients, which would be an indirect evidence of heavy and cumulated fluoroquinolone exposure with age among the general population in Taiwan. Control of fluoroquinolone consumption is mandatory to counteract the continuous increase of fluoroquinolone resistance.

Another worrisome finding was the diminished susceptibilities of carbapenems among isolates carrying both ESBL and AmpC β-lactamase genes. In our study, while the overall susceptibility to carbapenems for *K. pneumoniae* was high, the rates of susceptibility to ertapenem and imipenem was only 57.1% and 63%, respectively, in isolates positive for both ESBL and AmpC β-lactamase genes. Altered outer membranous permeability, such as porin mutation (OmpK35 or OmpK36 loss), coupled with the presence of ESBLs or AmpC β-lactamases, and the production of carbapenemases are two major mechanisms for carbapenem resistance in *K. pneumoniae* in Taiwan^27^,^31^. Since no carbapenemase-producers were detected in the present study, mutations in OmpK 35/36 plus carriage of ESBLs or AmpC β–lactamases are likely the major mechanism that led to the carbapenem resistance in our isolates.

In the present study, the predominant ESBL genes were CTX-M-type (64%), and most of AmpC β-lactamase genes belonged to DHA-type (90%). Previous studies had shown that CTX-M-type genes are currently the most common type of ESBL genes worldwide, and it is the same for community-sourced ESBL *K. pneumoniae* strains^7^,^20^. CMY-type gene was the most common AmpC β-lactamase gene among *Enterobacteriaceae* isolates in Asia-Pacific region, according to results from the SMART study^32^. However, several studies from Korea also demonstrated that DHA was the predominant type of AmpC β-lactamase gene among their *K. pneumoniae* isolates^12^,^18^. Together with our results, these results indicated a geographical distribution difference of endemic AmpC β-lactamase genes.

Phylogenetic analysis by MLST showed that ST11, ST48, ST15, and ST378 were the most common sequence types among our ESBL- and/or AmpC β-lactamase-producing *K. pneumoniae* isolates. However, these four major sequence types comprised only 52.2% of isolates, with 33.0% being ST11. A separate study focusing on imipenem-non-susceptible *K. pneumoniae* isolates during similar period in Taiwan also found ST11 and ST48 to be the dominant strains^33^. Globally, the epidemiological data on *K. pneumoniae* genotype is limited, but ST11 has been found to be one of the pandemic clones in Europe and Asia^34^,^35^. ST11 strains have also been associated with multi-drug resistance in a previous study^36^. Therefore, isolates of ST11 may be more prone to acquire resistance. The underlying mechanisms need further investigation. By PFGE, the ESBL and/or AmpC β-lactamase-producers showed a great diversity, and only one small cluster (15 isolates only) of ST11 isolates was found. However, in addition to having 3 three different combinations of ESBL and AmpC β-lactamase genes, only 2 of the 15 isolates in the cluster had indistinguishable PFGE patterns. Taken together, our molecular study results indicated that the increased prevalence of ESBL and/or AmpC-β-lactamase-producing *K. pneumoniae* was likely mostly due to transmission of the β-lactamase genes via horizontal gene transfer elements such as plasmids instead of clonal spread.

Using the 2014 CLSI breakpoints, 98.3% of our ESBL- and/or AmpC β-lactamase- positive isolates were non-susceptible to cefotaxime, while 84.5% of them were non-susceptible to ceftazidime. These findings are similar to our previous study on ESBL *E. coli*^37^. Cefotaxime was more sensitive than ceftazidime in identifying ESBL- and/or AmpC β-lactamase-producers in our *K. pneumoniae* isolates (98.3% vs. 84.5%). These results would be partly attributed to the predominance of CTX-M-type genes, the enzymes of which hydrolyze cefotaxime more efficiently than ceftazidime^38^.

The findings from our study have important clinical implications. It has been suggested that the empirical antibiotics therapy for severely infected patients should cover at least 90% of all possible bacterial pathogens^39^. Based on the present study, however, susceptibility to 3^rd^ generation cephalosporins and fluoroquinolones in our *K. pneumoniae* isolates was both below 90%, and these rates were even lower among isolates from elderly patients, at 81.2% for cefotaxime and 78.6% for ciprofloxacin. Besides, the prevalence of ESBLs-producers has reached 11.9% in 2012, indicating that 3^rd^ generation cephalosporins would no longer be reliable options for empirical therapy. Those antibiotics with higher susceptibility rates, especially carbapenems, to which over 95% of the isolates from all age groups were susceptible, would be more reasonable options for empirical therapy in critical conditions if *K. pneumoniae* infection, even from community settings, is suspected.

In the multivariate analysis, age, isolates from later study years, and isolates from urine were independent factors associated with ESBL-producing *K. pneumoniae*. Elderly patients might have more exposure to medical care, long-term care facilities, and antibiotics. Therefore, they would be at a higher risk of acquiring drug-resistant bacteria. As for AmpC β-lactamase-producers, recovery from urine was an independent factor. The reason that isolates from later study period was not an independent factor associated with AmpC β-lactamase-producers may be due to the limitation of statistical method itself. This is because no AmpC β-lactamase-producers were detected in TSAR III (2002), which led to a divergent estimation of regression coefficient for TSAR III (2002) in the multivariate regression model and in turn made the statistical result non-significant. However, by chi-square for trend analysis, isolates from later study period had significantly higher probability of being AmpC β-lactamase-producers. Therefore, we believe that isolates from later study period was a significant risk factor associated with AmpC β-lactamase-producers. The reasons for the strong association of urine isolates with ESBL- and/or AmpC β-lactamase-producers need further investigation.

Our study has several limitations. First, we have limited clinical information on the source patients. Therefore, we could not identify whether there were other independent factors, such as prior hospitalization, recent antibiotics use, and healthcare facility exposure, for ESBL- and/or AmpC β-lactamase-producers. However, age was an independent factor for ESBL-producers, thus prior hospitalization, and antibiotics as well as healthcare facility exposure were likely associated factors. Second, the isolates were collected biennially during a three-month period only. However, the enrolled isolates were from 25–28 hospitals over four geographical areas of Taiwan. Therefore, we consider that the results described here are representative for *K. pneumoniae* from community settings in Taiwan. Finally, the actual location of the ESBL and AmpC β-lactamase genes were not determined in our study. Although it has been shown that ESBL genes could reside on the chromosome^40^, several studies on ESBL-producing *K. pneumoniae* have found CTX-M-type and SHV-type ESBL genes to be mostly located on plasmids^41^,^42^,^43^,^44^. Furthermore, the great genotypic diversity of our ESBL and/or AmpC β-lactamase-producers, having different combinations of ESBL/AmpC β-lactamase genes even among isolates within the same PFGE cluster, implied that these resistant genes are acquired through transfer of plasmids among isolates of different genetic backgrounds.

In conclusion, our multicenter surveillance for *K. pneumoniae* isolates from community settings in Taiwan between 2002 and 2012 revealed decreased susceptibilities to most antibiotics, especially 3^rd^ generation cephalosporins and fluoroquinolones. The prevalence of ESBL- and AmpC β-lactamase-producers had also increased, indicating that the chance of encountering multidrug-resistant pathogens would increase even in the scenario of community-sourced infection. Co-resistance to other antibiotics, especially ciprofloxacin, among ESBL- and/or AmpC β-lactamase-producing *K. pneumoniae* is of particular concern since treatment options would be further limited. These results indicate that 3^rd^ generation cephalosporins may no longer be reliable empirical treatment choices, and further studies are needed to determine which antibiotics are current reasonable options when a patient presents with severe sepsis which might be caused by *K. pneumoniae*.

## Method

As part of the TSAR program, *K. pneumoniae* isolates were collected biennially from 2002 (TSAR III) to 2012 (TSAR VIII). During the collection year, isolates were collected from July to September from 25–28 regional hospitals and medical centers located in different geographical regions of Taiwan. Detailed collection protocol has been published previously^45^. All isolates were stored at −70 °C. In present study, we only enrolled *K. pneumoniae* clinical isolates from patients visiting emergency rooms or from outpatient clinics. Written informed consent was not obtained since all the isolates were recovered from clinical samples taken as part of standard care and the patient information was anonymized prior to analysis. TSAR program was approved by the Research Ethics Committee of National Health Research Institutes (NHRI), Taiwan (EC960205 and EC1010602-E), and was conducted in accordance with the principles of Declaration of Helsinki and the International Conference on Harmonization for Good Clinical Practice.

### Isolate identification

Isolates reported as *K. pneumoniae* were subcultured to blood agar and MacConkey agar plates for purity check. Species identification was confirmed at NHRI based on colony morphology, conventional biochemical reactions, and use of Vitek II GN cards (bioMérieux, Marcy l′Etoile, France).

### Antimicrobial susceptibility testing (AST)

Minimum inhibitory concentrations (MICs) were determined using reference broth microdilution method following the guidelines of the manufacturer and Clinical and Laboratory Standards Institute (CLSI) 2014^46^. Sensititre custom-designed plates were used from TSAR III (2002) to TSAR VI (2008), and the standard GNX2F plates were used in TSAR VII (2010) and TSAR VIII (2012) [ThermoFisher Scientific (formerly Trek Diagnostics), East Grinstead, UK]. All isolates were subcultured twice on sheep blood agar plates from −70 °C prior to AST. Quality control was performed each day using *Escherichia coli* ATCC 25922, *Escherichia coli* ATCC 35218, *Klebsiella pneumoniae* ATCC 700603, and *Pseudomonas aeruginosa* ATCC 27853.

The following agents were tested on isolates from all study years: amikacin, ampicillin, aztreonam, cefazolin, cefepime, cefotaxime, cefoxitin, ceftazidime, cefuroxime, ciprofloxacin, gentamicin, and imipenem. Other agents not tested in all years included (years tested) amoxicillin/clavulanate (2002–2008), ertapenem (2012), piperacillin (2002–2010), tetracycline (2002–2008), and tigecycline (2010 and 2012). Interpretive criteria are based on the 2014 CLSI breakpoints^46^. Susceptibility to tigecycline was interpreted using breakpoints proposed by the European Committee on Antimicrobial Susceptibilities Testing (EUCAST) (http://www.eucast.org/clinical_breakpoints/)

### Detections of ESBL, AmpC β-lactamase, and carbapenemase genes

The ESBL screening test-positive isolates were defined as isolates with aztreonam, ceftazidime, or cefotaxime MIC ≥ 2 mg/L according to the suggestions of CLSI^46^. The CLSI ESBL confirmatory test was performed on all isolates positive for ESBL screening test using cefotaxime and ceftazidime disks with and without clavulanate^46^. These ESBL screening test-positive isolates were also subject to detection of ESBL and/or AmpC β-lactamase genes by multiplex PCR using primers and following protocols that were previously described^47^,^48^. The DNA product of the *bla*_SHV_ gene was subject to NheІ restriction enzyme digestion to differentiate non-ESBL SHV and SHV-ESBL^49^. Isolates non-susceptible to carbapenem were tested by carbapenemase PCR using published methods^50^.

### Multi-locus sequence typing (MLST) and pulsed-field gel elelctrophoresis (PFGE)

Molecular typing of ESBL- and/or AmpC β-lactamase-producers was performed using MLST and PFGE following previously published protocols and information from the MLST website (http://bigsdb.pasteur.fr)^51^. For interpretation of the PFGE banding patterns, unweighted-pair group method using average linkages (UPGMA) dendrograms were constructed from the original data. Isolates that exhibited similarity of 80% or greater of their banding patterns were considered to belong to the same cluster if more than 3 isolates were present^52^.

### Data analysis

Susceptibility interpretation analysis was made using the WHONET software^53^. Duplicate isolates were excluded before analysis. TSAR III-V (2002–2006) and TSAR VI–VIII (2008–2012) were grouped as Period I and II, respectively, for the sake of comparison. Intermediate susceptibility and resistance were grouped together as ”non-susceptibility”. Categorical variables were compared using chi-square test or Fisher’s exact test (if the number was less than 10). If statistical difference was obtained after comparing categorical variables with three different levels, post-hoc analysis was then performed to identify which level was significantly different from the others. Trend analysis was made using chi-square. Multivariable logistic regression analysis was performed to assess the variables (including study year, specimen type, and patient age group) among ESBL- and/or AmpC β-lactamase-producers vs. non-producers. SAS 9.2 (SAS Institute, Cary, NC, USA) was used for the above analyses. A 2-tailed P value less than 0.05 was considered statistically significant.

## Additional Information

**How to cite this article**: Lin, W.-P. *et al*. The Antimicrobial Susceptibility of *Klebsiella pneumoniae* from Community Settings in Taiwan, a Trend Analysis. *Sci. Rep.* **6**, 36280; doi: 10.1038/srep36280 (2016).

**Publisher’s note:** Springer Nature remains neutral with regard to jurisdictional claims in published maps and institutional affiliations.

## Figures and Tables

**Table 1:** Drug susceptibilities (%) of *K. pneumoniae* by study periods.

Antimicrobial agents^1^	Period I (2002–2006) (n = 425)	Period II (2008–2012) (n = 591)	Total (n = 1016)	P value^2^
**β-lactams**				
Amoxicillin/clavulanate^3^	90.6	87.1	89.5	0.195
Aztreonam	93.2	88.5	90.5	0.012
Cefazolin	78.4	71.6	74.4	0.015
Cefuroxime	88.9	82.2	85.0	0.003
Cefoxitin	90.8	84.3	87.0	0.002
Cefotaxime	92.5	83.6	87.3	<0.001
Ceftazidime	94.1	85.8	89.3	<0.001
Cefepime	93.9	91.5	92.5	0.162
Ertapenem^3^	—	97.6	97.6	—
Doripenem^3^	—	99.2	99.2	—
Imipenem	98.6	96.8	97.5	0.067
Meropenem^3^	—	99.3	99.3	—
Piperacillin^3^	80.7	75.6	78.3	0.079
Piperacillin/tazobactam^3^	93.4	90.7	91.3	0.289
**Non β-lactams**				
Amikacin	96.2	93.6	94.7	0.062
Gentamicin^3^	87.7	82.2	84.5	0.019
Ciprofloxacin	89.9	81.6	85.0	<0.001
Levofloxacin^3^	—	80.8	80.8	—
Trimethoprim/sulfamethoxazole	77.7	74.6	75.9	0.267
Tetracycline^3^	76.9	72.6	75.6	0.248
Tigecycline^3^	—	97.8	97.8	—

^1^Susceptibility results are based on the 2014 CLSI breakpoints.

^2^P value for the comparison of susceptibilities between Period I and II.

^3^For amoxicillin/clavulanate, all isolates from Period I were tested but only 186 isolates from Period II were tested. For doripenem, ertapenem, and meropenem, only 250, 210, and 405 isolates from Period II, respectively, were tested. For piperacillin, all isolates from Period I and 381 isolates from Period II were tested. For piperacillin/tazobactam, 152 isolates from Period I and all isolates from Period II were tested. For tetracycline, all isolates from Period I and 186 isolates from Period II were tested. For tigecycline, only 405 isolates from Period II were tested.

**Table 2:** Drug susceptibilities (%) of *K. pneumoniae* by specimen types and age groups.

Antimicrobial agents^1^	Specimen types			Age groups		
	Blood (n = 378)	Urine (n = 309)	Others (n = 329)	Adult (n = 450)	Elderly (n = 490)	Pediatric (n = 56)
**β-lactams**						
Amoxicillin/clavulanate	92.0	80.52,^3^	95.4	94.8	83.0^4^	90.7
Aztreonam	92.9	83.32,^3^	95.2	94.4	85.7^4^,^5^	96.4
Cefazolin	79.1	59.92,^3^	84.1	81.8	66.3^4^	80.4
Cefazolin_urine	—	84.1	—	—	—	—
Cefuroxime	89.4	75.12,^3^	90.3	89.8	79.4^4^	92.9
Cefoxitin	91.0	79.02,^3^	90.6	91.1	82.0^4^,^5^	94.6
Cefotaxime	90.5	79.3^2^,^3^	91.9	92.4	81.2^4^,^5^	94.6
Ceftazidime	92.1	82.4^2^,^3^	93.2	93.1	84.3^4^,^5^	98.2
Cefepime	94.7	85.7^2^,^3^	97.1	96.0	88.6^4^	96.4
Ertapenem	98.9	97.1	95.7	98.8	96.6	100
Doripenem	100	98.8	98.2	100	98.6	100
Imipenem	97.9	95.73	99.0	98.0	96.9	98.2
Meropenem	99.4	99.3	98.9	100	98.7	100
Piperacillin	82.5	63.3^2^,^3^	88.6	84.7	71.2^4^	79.2
Piperacillin/tazobactam	94.6	84.4^2^,^3^	94.6	94.5	88.0^4^	97.1
**Non β-lactams**						
Amikacin	95.2	91.8^3^	97.1	96.7	92.5	96.4
Gentamicin	87.2	74.2^2,^^3^	92.2	90.0	78.5^4^	89.1
Ciprofloxacin	89.2	73.0^2^,^3^	92.9	90.2	78.6^4^,^5^	98.2
Levofloxacin	88.9	66.3^2^,^3^	87.5	86.9	76.2	87.5
Trimethoprim/sulfamethoxazole	79.6	60.8^2^,^3^	87.4	83.1	68.2^4^	80.4
Tetracycline	76.0	63.6^2^,^3^	86.1	79.8	70.5^4^	72.1
Tigecycline	97.2	97.8	98.9	98.2	97.4	100

P.S.1 For amoxicillin/clavulanate, only 611 isolates (200 from blood, 195 from urine, and 216 from other specimens; 287 from adult, 264 from elderly, and 43 from pediatric; 14 with missing age data) were tested. For doripenem, only 250 isolates (108 from blood, 86 from urine, and 56 from other specimens; 99 from adult, 143 from elderly, and 8 from pediatric) were tested. For ertapenem, only 210 isolates (93 from blood, 70 from urine, and 47 from other specimens; 83 from adult, 119 from elderly, and 8 from pediatric) were tested. For meropenem, only 405 isolates (178 from blood, 134 from urine, and 93 from other specimens; 163 from adult, 226 from elderly, and 13 from pediatric; 3 with missing age data) were tested. For piperacillin, 806 isolates from Period II (285 from blood, 259 from urine, and 262 from other specimens; 367 from adult, 371 from elderly, and 48 from pediatric; 20 with missing age data) were tested. For piperacillin/tazobactam, 743 isolates (294 from blood, 244 from urine, and 205 from other specimens; 307 from adult, 391 from elderly, and 35 from pediatric; 10 with missing age data) were tested. For tigecycline, only 405 isolates (178 from blood, 134 from urine, and 93 from other specimens; 163 from adult, 226 from elderly, and 13 from pediatric; 3 with missing age data) were tested. For tetracycline, 611 isolates (200 from blood, 195 from urine, and 216 from other specimens; 287 from adult, 264 from elderly, and 43 from pediatric; 17 with missing age data) were tested.

^1^Susceptibility results are based on the 2014 CLSI breakpoints. Overall, there were 20 patients with missing data on their age.

^2^The rates of susceptibility to these agents were significantly lower (P < 0.05) among urine isolates compared with blood isolates.

^3^The rates of susceptibility to these agents were significantly lower (P < 0.05) among urine isolates compared to isolates from other specimen types.

^4^The rates of susceptibility to these agents were significantly lower (P < 0.05) among isolates from elderly patients than those from adults.

^5^The rates of susceptibility to these agents were significantly lower (P < 0.05) among isolates from elderly patients compared with those from the pediatric patients.

**Table 3:** Susceptibilities (%) to key agents among the *K. pneumoniae* isolates with/without ESBL and/or AmpC β-lactamase genes (138 isolates were screened for these genes).

Antimicrobial agents^1^	Combination of ESBL/AmpC β-lactamase genes			
	+/+ (n = 27)	+/− (n = 54)	−/+ (n = 34)	−/− (n = 901)
Amikacin^2^,^3^,^4^	25.9	64.8	67.7	99.6
Aztreonam^2^,^3^,^4^	0	18.5	44.1	99.2
Cefotaxime^2^,^3^,^4^	0	0	5.9	98.2
Ceftazidime^2^,^3^,^4^	0	27.8	8.8	98.7
Cefepime (S/SDD)^2^,^3^,^4^,^5^	25.9/3.7	14.8/22.2	88.2/8.8	99.3/0.6
Ciprofloxacin^2^,^3^,^4^	0	33.3	38.2	92.5
Ertapenem^2^,^3^,^4^	57.1 (n = 7)	94.4 (n = 17)	92.3 (n = 12)	100 (n = 172)
Imipenem^2^,^4^	63.0	96.3	88.2	99.0

^1^Susceptibility results are based on the 2014 CLSI criteria.

^2^Significant lower rates of susceptibility (P < 0.001) in isolates positive for both ESBL and AmpC β-lactamase genes than those negative for both ESBL and AmpC β-lactamase genes.

^3^Significantly lower susceptible rate (P < 0.001) between isolates only positive for ESBL genes and those negative for both ESBL and AmpC β-lactamase genes.

^4^Significantly lower susceptible rate (P < 0.001) between isolates only positive for AmpC β-lactamase genes and those negative for both ESBL and AmpC β-lactamase genes.

^5^Cefepime results ar shown in susceptible and susceptible dose dependent (SDD) categories.

**Table 4:** Chi-square for trend analysis on the prevalence of ESBLs, AmpC β-lactamases, and cefotaxime non-susceptibility.

	TSAR III	TSAR IV	TSAR V	TSAR VI	TSAR VII	TSAR VIII	P
ESBLs	6/124	6/149	14/152	16/186	14/195	25/210	0.012
AmpC β-lactamases	0/124	5/149	6/152	12/186	18/195	20/210	<0.001
ESBLs + AmpC β-lactamses	0/124	2/149	4/152	8/186	6/195	7/210	0.039
Cefotaxime non-susceptibility	7/124	9/149	16/152	21/186	37/195	39/210	<0.001

**Table 5:** Univariate and multivariate analysis for factors associated with carriage of ESBL and AmpC β-lactamase genes in *K. pneumonia.*

Variables	ESBL genes				AmpC β-lactamase genes			
	Univariate Analysis		Multivariate analysis		Univariate Analysis		Multivariate analysis	
	Odds ratio	P	Odds ratio (95% C.I.)	P	Odds ratio	P	Odds ratio (95% C.I.)	P
Study year (using TSAR VIII [2012] as baseline)								
TSAR III (2002)	0.38	0.037	0.47 (0.18–1.21)	NS	<0.001	NS	<0.001 (<0.001–>999.999)	NS
TSAR IV (2004)	0.31	0.013	0.37 (0.15–0.95)	0.040	0.33	0.030	0.37 (0.13–1.03)	NS
TSAR V (2006)	0.75	NS	0.81 (0.40–1.66)	NS	0.39	0.049	0.39 (0.15–1.02)	NS
TSAR VI (2008)	0.70	NS	0.80 (0.40–1.59)	NS	0.66	NS	0.71 (0.33–1.52)	NS
TSAR VII (2010)	0.57	NS	0.57 (0.28–1.16)	NS	0.97	NS	0.98 (0.50–1.95)	NS
Age groups (using pediatric patients as baseline)								
Adult patients	1.07	NS	1.43 (0.40–5.07)	NS	1.19	NS	1.31 (0.28–6.04)	NS
Elderly patients	3.33	0.047	3.51 (1.05–11.78)	0.042	3.74	NS	3.17 (0.74–13.69)	NS
Specimen types (using urine as baseline)								
Blood	0.34	<0.001	0.33 (0.19–0.57)	<0.001	0.33	<0.001	0.31 (0.16–0.59)	<0.001
Others	0.21	<0.001	0.25 (0.13–0.50)	<0.001	0.38	0.004	0.49 (0.25–0.97)	0.039
